# Global, regional, and national burden and quality of care index (QCI) of thyroid cancer: A systematic analysis of the Global Burden of Disease Study 1990–2017

**DOI:** 10.1002/cam4.3823

**Published:** 2021-03-05

**Authors:** Sina Azadnajafabad, Sahar Saeedi Moghaddam, Esmaeil Mohammadi, Negar Rezaei, Erfan Ghasemi, Nima Fattahi, Arya Aminorroaya, Reza Azadnajafabad, Armin Aryannejad, Nazila Rezaei, Shohreh Naderimagham, Vahid Haghpanah, Ali H. Mokdad, Hossein Gharib, Farshad Farzadfar, Bagher Larijani

**Affiliations:** ^1^ Non‐Communicable Diseases Research Center Endocrinology and Metabolism Population Sciences Institute Tehran University of Medical Sciences Tehran Iran; ^2^ Endocrinology and Metabolism Research Center, Endocrinology and Metabolism Clinical Sciences Institute Tehran University of Medical Sciences Tehran Iran; ^3^ Department of Electrical Electronic and Information Engineering University of Bologna Bologna Italy; ^4^ Institute for Health Metrics and Evaluation University of Washington Seattle WA USA; ^5^ Mayo Clinic College of Medicine Rochester MN USA

**Keywords:** gender disparity, global burden of disease, healthcare quality, quality of care index, socioeconomic factors, thyroid cancer

## Abstract

**Background:**

Thyroid cancer (TC) is the most prevalent malignancy of the endocrine system. Over the past decades, TC incidence rates have been increasing. TC quality of care (QOC) has yet to be well understood. We aimed to assess the quality of TC care and its disparities.

**Methods:**

We retrieved primary epidemiologic indices from the Global Burden of Disease (GBD) 1990–2017 database. We calculated four secondary indices of mortality to incidence ratio, disability‐adjusted life years (DALYs) to prevalence ratio, prevalence to incidence ratio, and years of life lost (YLLs) to years lived with disability (YLD) ratio and summarized them by the principal component analysis (PCA) to produce one unique index presented as the quality of care index (QCI) ranged between 0 and 100, to compare different scales. The gender disparity ratio (GDR), defined as the QCI for females divided by QCI for males, was applied to show gender inequity.

**Results:**

In 2017, there were 255,489 new TC incident cases (95% uncertainty interval [UI]: 245,709–272,470) globally, which resulted in 41,235 deaths (39,911–44,139). The estimated global QCI was 84.39. The highest QCI was observed in the European region (93.84), with Italy having the highest score (99.77). Conversely, the lowest QCI was seen in the African region (55.09), where the Central African Republic scored the lowest (13.64). The highest and lowest socio‐demographic index (SDI) regions scored 97.27 and 53.85, respectively. Globally, gender disparity was higher after the age of 40 years and in favor of better care in women.

**Conclusion:**

TC QOC is better among those countries of higher socioeconomic status, possibly due to better healthcare access and early detection in these regions. Overall, the quality of TC care was higher in women and younger adults. Countries could adopt the introduced index of QOC to investigate the quality of provided care for different diseases and conditions.

## Graphical abstract

In this study, we retrieved primary epidemiologic data from the Global Burden of Disease (GBD) 1990–2017 database. We produced one unique index, presented as the quality of care index (QCI), to compare different geographical, socio‐developmental, and age scales. The main finding of this survey was the better quality of care among those of higher socioeconomic status, revealing the effect of better healthcare access and early detection in these regions. Also, quality of thyroid cancer care was better in women and younger adults. Evaluation and comparison of thyroid cancer care among various regions and populations, provides data for policy‐makers and healthcare authorities to improve quality of care for patients with thyroid cancer in different settings.

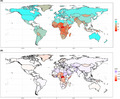

## 1 INTRODUCTION

Thyroid cancer (TC) is the most common cancer of the endocrine system worldwide and mostly affects young adults.^1^ Estimates show that TC prevalence is 1%–5% in women and 2% in men. It is the 7th and 14th most common cancer in women and men, respectively.^2^ Several reports have described a significant increase in TC incidence over the recent decades.^3^, ^4^ Based on this increasing trend, TC will replace colorectal cancer as the fourth leading cancer by 2030, followed by breast, prostate, and lung cancers.^4^, ^5^, ^6^, ^7^ Thus, such an increase will cause immense clinical and economic burdens that must be considered.^8^

Various investigations suggest multiple TC incidence disparities, such as age, gender, education, race, and socioeconomic status.^9^, ^10^ For example, TC incidence is 2–3 times higher in women, suggesting the role of sex steroids in TC carcinogenesis.^11^, ^12^ Also, TC incidence is higher in more developed countries. Nevertheless, the mortality rate is higher in less developed countries.^2^, ^13^ Regardless of all of these disparities, it is essential to properly prioritize resources to alleviate the effects of this cancer's increasing incidence.^8^ Quality of care (QOC) means providing patients with timely services by adopting professional skills and knowledge to achieve the required health outcomes.^14^ As investigated earlier, the disparity in cancer QOC is a major problem in healthcare systems.^15^, ^16^ The debate about QOC and its components has always been a primary epidemiologic concern but less demonstrated. However, previous studies utilized indices like mortality to incidence ratio (MIR) to assess QOC in different cancers.^17^, ^18^, ^19^

In this study, we introduced a new quality of care index (QCI) for TC and utilized it to compare different regions, age groups, and genders. The application of this index would help to discuss care quality, better understand the controversies in different studies, the real burden of TC, and its impact on health services and communities. Finally, it may be applied to help effective policymaking, resource allocation and research funding.

## 2 MATERIALS AND METHODS

### 2.1 Overview and data resources

In this study, we utilized the Global Burden of Disease (GBD) data from 1990 to 2017, available on the Institute for Health Metrics and Evaluation (IHME) website. The data can be accessed on the GBD‐compare webpage in the “Causes” section under the GBD code “B.1.23”.^20^ In the 10th revision of the International Classification of Diseases (ICD‐10) system, TC is documented as a malignant neoplasm of the thyroid gland under diagnosis codes C73 and Z85.850 for incidence data of TC and codes C73–C73.9, D09.3, D09.8, D34–D34.9, and D44.0 for TC mortality data.^21^ This study was designed and conducted according to the GATHER guidelines (guidelines for accurate and transparent health estimates reporting).^22^

### 2.2 IHME‐GBD methods summary

IHME provides GBD data through a systematic approach to global, regional, national, and other categories of countries and regions to describe epidemiologic data on various diseases, risk factors, and injuries stratified by sex, age, and geographical categories. Focusing on cancer data provided by IHME, multiple cancer registry databases are gathered, and estimations are made on a compilation of data. Two essential databases for estimations on cancers are cancer incidence data sources and cancer mortality data sources.^7^, ^23^ Estimations and adjustments on data are conducted by various modelings like the Cause of Death Ensemble model (CODEm) that explores a large number of possible models to estimate trends in causes of death, CoDCorrect that is responsible for making the Causes of Death and All‐cause Mortality results internally consistent, and DisMod‐MR 2.1 to model prevalence data of causes.^23^, ^24^ Results of the estimated data of causes in multiple levels are then provided to explore the burden of diseases.

### 2.3 Quality of care index

To assess the QOC parameters in this study, four secondary indices were generated; MIR (#1), disability‐adjusted life years (DALY) to prevalence ratio (#2), prevalence to incidence ratio (#3), and years of life lost (YLL) to years lived with disability (YLD) ratio (#4).

$$\left(\left(\#1\right)\right)\;\mathrm{MIR}\;\left(\left(\mathrm{Mortality}\;\mathrm{to}\;\mathrm{Incidence}\;\mathrm{Ratio}\right)\right)=\frac{\mathrm{Mortality}}{\mathrm{Incidence}},$$

$$\left(\left(\#2\right)\right)\;\mathrm{DALY}\;\mathrm{to}\;\mathrm{Prevalence}\;\mathrm{Ratio}=\frac{\mathrm{DALY}\;}{\mathrm{Prevalence}\;},$$

$$\left(\left(\#3\right)\right)\;\mathrm{Prevalence}\;\mathrm{to}\;\mathrm{Incidence}\;\mathrm{Ratio}=\frac{\mathrm{Prevalence}\;}{\mathrm{Incidence}},$$

$$\left(\left(\#4\right)\right)\;\mathrm{YLL}\;\mathrm{to}\;\mathrm{YLD}\;\mathrm{Ratio}=\frac{\mathrm{YLL}\;}{\mathrm{YLD}\;}.$$

Each mentioned index was produced to consider an aspect of TC QOC. The first ratio (MIR) takes into account that considering a stable incidence of TCs in a geographical region, higher mortality values pertain to worse care provided to these patients. It is believed that whenever a new TC case emerges in a population, averting his/her death is a promise of the health system. The second ratio indicates that in cases of a similar prevalence of TCs in different regions, higher DALYs are considered as worse care quality. The third ratio is interpreted as in regions with similar incidence rates of TC, a higher prevalence of its premise that patients are managed more accordingly and their deaths are prevented. The fourth ratio's higher values represent worse conditions since the low QOC in a region results in higher YLLs and fewer YLDs (patients are ceased earlier than their mean‐expected life years). This ratio assumes that living with a disability related to TC is superior to dying in advance. This ratio also highlights the effectiveness of the health system to postpone patients’ deaths. Since these indices were calculated for each age group with TC separately, the impact of life expectancy and competing risks of death are omitted from the estimations.

To sum up these indices into one unique index, we used the principal component analysis (PCA) method. PCA is a multivariate statistical technique that obtains linear combinations of various data sets.^25^ The first component is a linear combination of all variables that explains these variables the best. We considered the QCI as the first component derived from PCA. The QCI scores were computed and rescaled into 0–100 scales, where higher scores indicated better QOC. Our analysis on PCA of the four secondary indices showed an excellent proportion of 95.12% of variance explained by the first component.

We calculated the QCI and socio‐demographic index (SDI) quintiles on a global scale based on the WHO regions; European region, region of the Americas, Western Pacific region, Eastern Mediterranean region, South East Asia region, and African region. We focused mainly on the QCI results of 2017 due to recent advances in the TC care and to investigate the present status of TC care in different scales. The SDI is a concise measure of socio‐demographic development used in GBD studies. “It is the composite average of rankings based on average income per capita, educational attainment, and total fertility rate”.^26^ The SDI quintiles are classified as high, high‐middle, middle, low‐middle, and low. It must be noted that the calculated QCI for each scale is a compilation of the 28‐year study period's data of that scale. Details on the calculation and codes of QCI are available in a published protocol by authors of this research center for further examination and utilization for other conditions and diseases.^27^ Also, this index is validated for other cancers which results are published elsewhere.^28^, ^29^

### 2.4 Age and gender disparity

In this study, we classified age in 5‐year intervals. To analyze and report the four above‐mentioned primary measures, we used age‐standardized measures as rates for 100,000 population. QCI for each age group was calculated in different scales such as global and SDI quintiles. Furthermore, trends depict the disparity of care in different age groups.

To evaluate the gender disparity of care in TC, we generated a gender disparity ratio (GDR), which is calculated by dividing the QCI score for females by the QCI score for males.

$$\mathrm{GDR}=\frac{\;QCI\;for\;Females}{\;QCI\;for\;Males}.$$

We then calculated this ratio for a global scale, SDI quintiles, and all countries. Near‐one ratio values showed the least disparity of care between the two sexes. Values higher or lower than one indicated a disparity of care in favor of one of the genders. However, TC prevalence may be different in the two sexes. Furthermore, equity in QOC among both genders is substantial, and a value equal to one indicated this equity. One of the limitations of this comparison, where various countries were to be compared, was that two countries with similar near‐one ratios had conflicting QCI scores for the two sexes. Therefore, GDR was interpreted with caution. To correct this limitation, we made a scatter plot that showed QCIs for both sexes, making it possible to compare different countries by their QCIs.

### 2.5 Further validation of QCI

Trying to introduce a new method of evaluating QOC in TC in this survey, the developed index results are comparable to a previous study that presented the healthcare access and quality index (HAQI) assessing the QOC in various diseases.^30^ Regarding estimation of the correlation between QCI and HAQI, a mixed effect model utilized QCI as the dependent variable and independent variables including inpatient and outpatient healthcare utilization, mortality and prevalence of TC, and body mass index (BMI) as the only available estimated risk factor of TC in GBD Compare were included.^20^ The estimated correlation from the model for TC was 0.80, indicating a remarkable association of two indices and validating QCI as an applicable measure of the QOC in TC patients.

### 2.6 Decomposition analysis

Due to the importance of investigating the increasing TC incidence trend, we did a further decomposition analysis of incidence trend 1990–2017 for TC. Three significant contributors to the incidence trend are population growth, population aging, and an increase in age‐specific rates of TC incidence. Decomposition analysis holds two of these three contributors constant and investigates the third one's contribution to a trend change.^7^ Expected TC incident cases in 2017 were estimated using two hypothetical demographic scenarios: first, applying the age‐specific rates of TC incidence in 1990 into 2017 population size; second, applying the age structure and age‐specific TC incidence rates in 1990 into the 2017 population size and structure. The difference between the two scenarios was considered as the contribution of population aging. Differences between the second scenario and TC incidences in 2017 were considered as the contribution of population growth. The remaining increase in TC incidence was considered as the changes in the age‐specific rates of TC incidence.^31^ Contributions of these three factors were reported as the percent of change and the overall impact of three of them in global and SDI scales.

### 2.7 Statistical analysis

Primary index values were reported with a 95% uncertainty interval (UI) of all‐age numbers and age‐standaridized rates per 100,000 population. The estimation and trend of changes were considered as significant when the UIs did not overlap over time. The PCA method applied to generate QCI was mentioned earlier. All the statistical analyses, plots, and numbers created in this study were performed by R for windows v 3.6.1 and RStudio v 1.0.136 (http://www.r‐project.org/, RRID: SCR_001905).^32^

## 3 RESULTS

### 3.1 Incidence, mortality, DALYs, and other epidemiologic indices of TC

The results revealed significant numbers and changes in TC during the study period. In 1990, the estimated global TC incidence was about 95,026 new cases (95% UI: 90068–100724) for all ages. In 2017, this number increased to 255,489 new cases (245,709–272,471). A comparison of these numbers showed a 28‐year percent change of about 169% increase in the number of Incidences. The number of deaths in 1990 was 22,065 (20,812–24,216), while in 2017, this number had increased to 41,235 (39,911–44,139). A similar change in this index happened over time, which was an 87% increase in deaths. In 1990, the DALYs for TC were estimated at 648,235 years (595,575–713,243). In 2017, the DALYs were 1,133,175 years (1,073,443–1,227,486). A complete list of these indices is available in Table 1. Among the different SDI regions, high SDI regions had the highest (5.17), and low SDI regions had the lowest (1.63) incidence rates of all ages per 100,000 population. In contrast, the mortality rate was highest in low SDI regions (0.62) and lowest in high and high–middle SDI regions (0.44). Further details about these indices can be seen in Table S1.

**TABLE 1:** Global trend of primary indices with 95% uncertainty interval (UI) of thyroid cancer for all‐ages numbers and age‐standardized rates, for each and both sexes, in 1990 and 2017 and percent of changes

Year	Sex	Prevalence		Incidence		Deaths		DALYs (disability‐adjusted life years)		YLLs (years of life lost)		YLDs (years lived with disability)	
Metric		Number (95% UI)	Rate (95% UI)	Number (95% UI)	Rate (95% UI)	Number (95% UI)	Rate (95% UI)	Number (95% UI)	Rate (95% UI)	Number (95% UI)	Rate (95% UI)	Number (95% UI)	Rate (95% UI)
1990 estimate	Both	772,090 (730,078–816,604)	16.85 (16.02–17.79)	95,026 (90,068–100,724)	2.11 (2.01–2.24)	22,065 (20,812–24,216)	0.55 (0.52–0.60)	648,235 (595,575–713,243)	14.44 (13.35–15.85)	599,107 (551,421–655,986)	13.34 (12.38–14.57)	49,129 (34,036–67,282)	1.10 (0.77–1.49)
	Female	585,224 (546,515–628,350)	24.93 (23.34–26.74)	70,852 (66,088–76,423)	3.04 (2.85–3.28)	14,509 (13,461–16,775)	0.66 (0.61–0.76)	421,306 (375,691–489,007)	17.94 (16.11–20.85)	385,301 (345,058–445,429)	16.39 (14.77–18.97)	36,005 (24,735–49,252)	1.55 (1.07–2.12)
	Male	186,866 (180,378–194,445)	8.56 (8.28–8.89)	24,174 (23,329–25,213)	1.15 (1.11–1.19)	7,557 (72,15–81,50)	0.41 (0.40– 0.44)	226,929 (213,366–247,064)	10.63 (10.05–11.51)	213,806 (201,789–233,643)	10.00 (9.49–10.86)	13,124 (9,199–17,391)	0.63 (0.45–0.83)
2017 estimate	Both	2,144,939 (2,059,508–2,287,830)	26.37 (25.32–28.11)	255,489 (245,709–272,471)	3.15 (3.03–3.36)	41,235 (39,911–44,139)	0.52 (0.51–0.56)	1,133,175 (1,073,443–1,227,486)	14.08 (13.34–15.27)	1,001,180 (963,576–1,073,978)	12.45 (11.97–13.36)	131,995 (91,685–180,822)	1.63 (1.13–2.23)
	Female	1,530,875 (1,451,954–1,667,069)	37.10 (35.16–40.41)	179,401 (170,401–195,543)	4.34 (4.12–4.73)	24,076 (23,057–26,832)	0.57 (0.54–0.63)	664,929 (618,498–746,730)	16.03 (14.89–17.98)	573,957 (543,198–642,884)	13.83 (13.06–15.47)	90,972 (62,469–125,992)	2.20 (1.51–3.05)
	Male	614,063 (585,561–640,775)	15.45 (14.72–16.12)	76,088 (72,576–79,289)	1.94 (1.86–2.02)	17,159 (16,412–17,772)	0.47 (0.45–0.49)	468,246 (444,424–492,880)	12.06 (11.45–12.69)	427,223 (407,301–443,033)	11.01 (10.49–11.41)	41,024 (28,974–55,544)	1.05 (0.74–1.42)
28‐year percent change %	Both	+177.81 (161.41–200.57)	+56.46 (47.50–68.66)	+168.86 (153.47–190.04)	+49.20 (40.88–60.16)	+86.88 (75.62–99.73)	−4.41 (−9.77 to 1.70)	+74.81 (61.03–92.65)	−2.50 (−9.80 to 6.64)	+67.11 (53.93–84.47)	−6.70 (−13.70 to 2.19)	+168.67 (152.47–189.08)	+48.51 (39.72–59.28)
	Female	+161.59 (140.54–187.86)	+48.84 (37.05–63.10)	+153.21 (132.85–177.57)	+42.62 (31.45–55.76)	+65.94 (52.09–79.70)	−13.69 (−20.74 to −6.88)	+57.83 (40.04–79.77)	−10.64 (−20.58 to 1.26)	+48.96 (32.11–70.30)	−15.62 (−24.88 to −4.03)	+152.67 (131.37–177.41)	+42.11 (30.14–55.37)
	Male	+228.61 (204.72–246.68	80.50 (67.63–89.99)	+214.75 (191.13–231.94)	+69.36 (57.10–78.06)	+127.06 (103.93–141.68)	+15.09 (4.40–21.83)	+106.34 (82.56–122.57)	+13.45 (0.88–21.68)	+99.82 (76.34–115.46)	10.09 (−2.19 to 18.10)	+212.59 (187.87–232.28)	+66.81 (53.59–77.59)

Note.

Values are reported for all ages and rates are per 100,000 population.

A further investigation in contributors to the increasing trend of TC incidence revealed a higher contribution of increase in age‐specific incidence rates of TC globally and SDI quintiles, for both and each sex. Globally, population growth had 51.21%, population aging had 28.74%, and age‐specific incidence increase had 88.91% proportion contributing to an overall increase of 168.86% in TC incidence estimated for both sexes. Also, decomposition results showed a higher overall increase in TC incidence in males than females (Table 2).

**TABLE 2:** Global and socio‐demographic index (SDI) regional decomposition analysis of thyroid cancer incidence trend, 1990–2017

Region	Sex	Number of incidences		Expected number of incidences in 2017		Causes of incidences change trend 1990–2017			
		1990	2017	Population growth	Population growth and aging	Population growth	Population aging	Age‐specific incidence rate	Overall
Global	Both	95,026	255,489	143,690	171,003	51.21%	28.74%	88.91%	168.86%
	Female	70,852	179,401	107,541	126,227	51.78%	26.37%	75.05%	153.21%
	Male	24,174	76,088	36,416	44,676	50.64%	34.17%	129.94%	214.75%
High SDI	Both	20,986	58,681	28,612	36,291	22.07%	24.93%	58.02%	105.03%
	Female	16,243	41,298	21,891	27,384	21.42%	20.16%	47.42%	89.01%
	Male	4,743	17,383	6,541	8,492	22.76%	32.58%	89.37%	144.71%
High–middle SDI	Both	42,954	88,070	52,436	63,147	36.34%	36.59%	106.69%	179.63%
	Female	30,597	57,831	37,153	43,321	34.77%	33.81%	85.66%	154.25%
	Male	12,357	30,238	15,169	19,195	37.93%	41.12%	187.49%	266.54%
Middle SDI	Both	9,649	31,536	16,993	18,513	48.29%	46.68%	198.96%	293.93%
	Female	7,612	24,653	13,609	14,869	50.19%	46.19%	170.40%	266.78%
	Male	2,036	6,883	3,534	3,890	46.43%	51.63%	283.64%	381.70%
Low–middle SDI	Both	5,713	15,404	11,398	11,743	76.12%	15.76%	134.98%	226.85%
	Female	4,390	11,741	8,892	9,208	78.78%	16.55%	128.53%	223.86%
	Male	1,323	3,663	2,600	2,657	73.52%	17.48%	147.02%	238.02%
Low SDI	Both	15,468	60,932	22,937	30,157	99.51%	6.04%	64.08%	169.63%
	Female	11,814	43,332	17,743	23,200	102.55%	7.20%	57.69%	167.44%
	Male	3,654	17,600	5,350	7,237	96.58%	4.31%	76.01%	176.90%

### 3.2 Quality of care index

**FIGURE 1:**
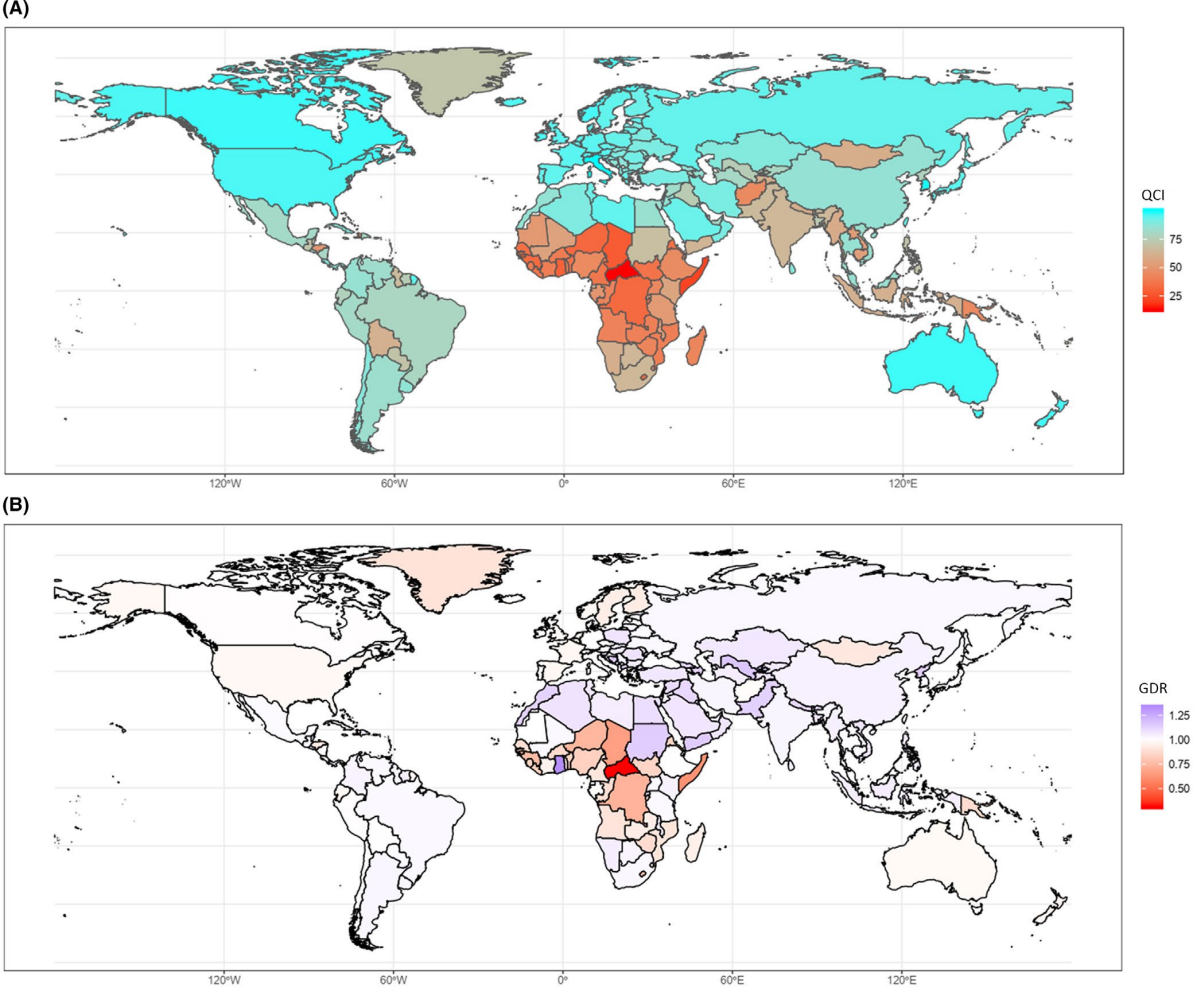
(A) Global distribution of estimated quality of care index (QCI). (B) Global distribution of estimated gender disparity ratio (GDR) based on QCI.

**FIGURE 2:**
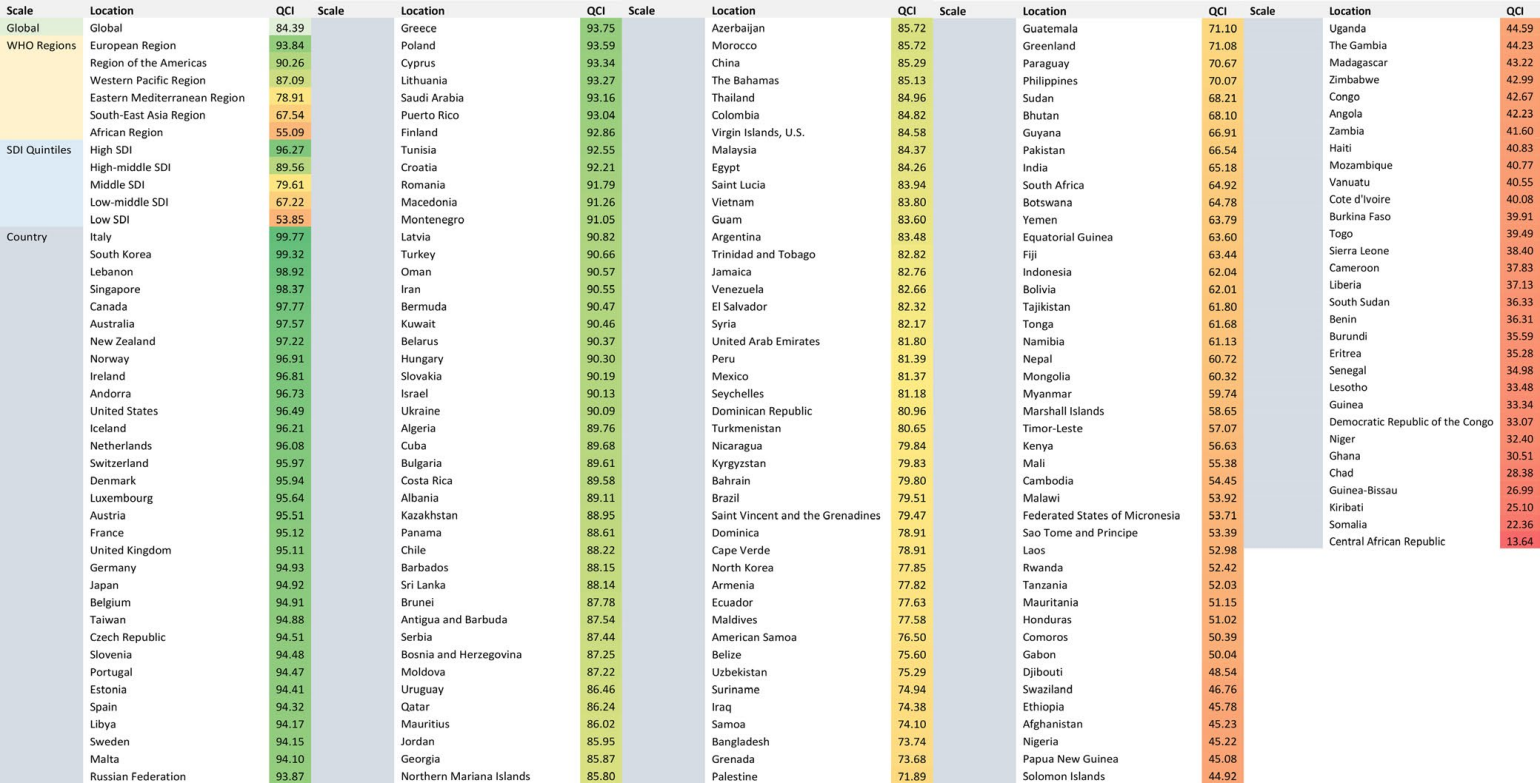
Detailed numbers of estimated quality of care index (QCI) in global, regional, socio‐demographic index (SDI) quintiles scales, and for 195 countries in 2017.

The estimated overall global QCI score was 84.39. The QCI scores for each WHO region were 93.84 for the European region, 90.26 for the region of the Americas, 87.09 for the Western Pacific region, 78.91 for the Eastern Mediterranean region, 67.54 for the South East Asia region, and 55.09 for the African region. The top five countries with the highest QCI scores were Italy (99.77), South Korea (99.32), Lebanon (98.92), Singapore (98.37), and Canada (97.77). On the other hand, the five countries with the lowest QCI scores were Chad (28.38), Guinea‐Bissau (26.99), Kiribati (25.10), Somalia (22.36), and the Central African Republic (13.64). The QCI scores in different SDI quintiles were 96.27 for high SDI, 89.56 for high–middle SDI, 79.61 for middle SDI, 67.22 for low–middle SDI, and 53.85 for low SDI. Figure 1A illustrates the global view of QCI distribution, while the details are presented in Figure 2.

### 3.3 Age disparity

**FIGURE 3:**
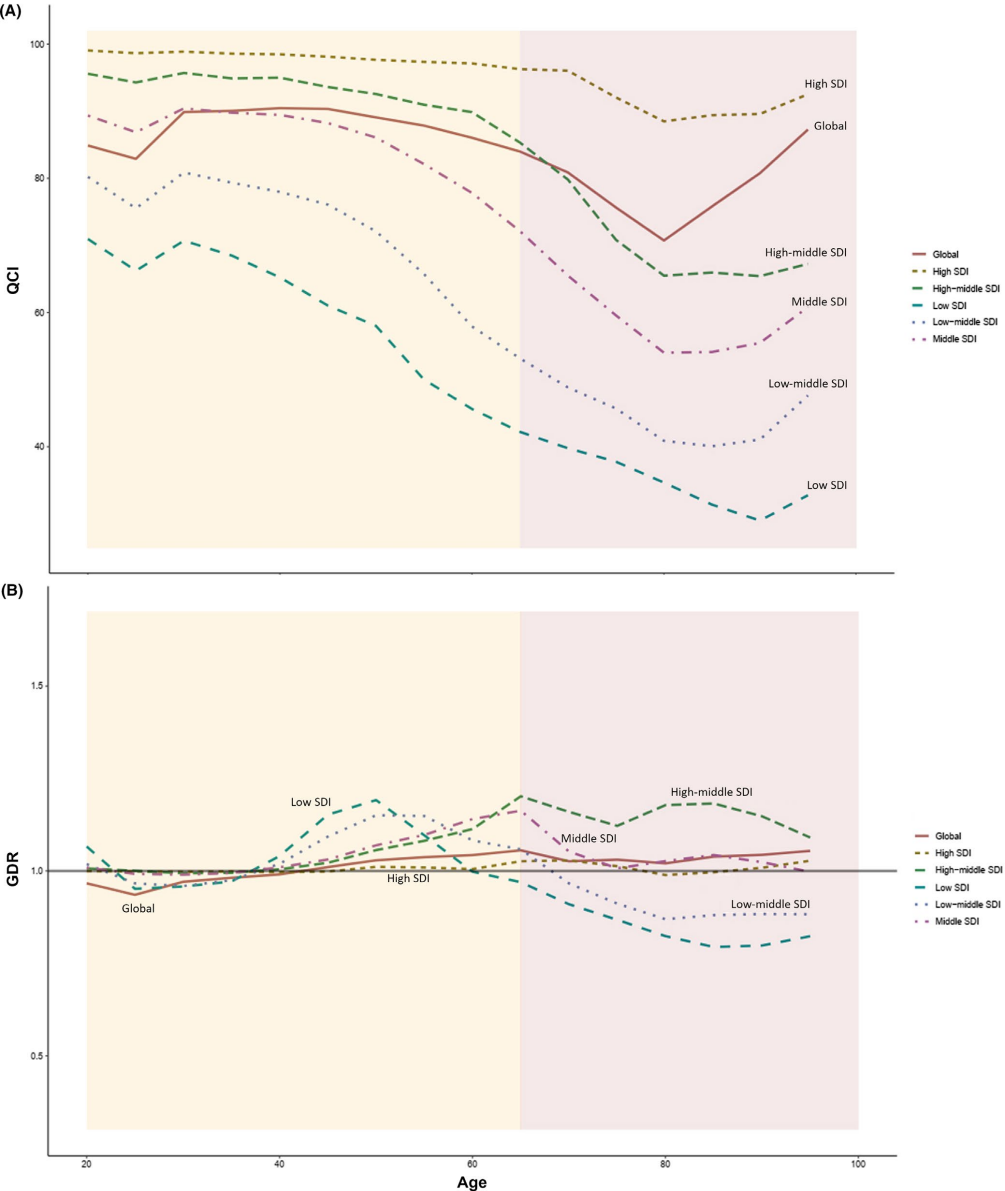
(A) Trend of estimated quality of care index (QCI) in five different socio‐demographic index (SDI) quintiles in comparison to global trend and in different age categories. B. Trend of estimated gender disparity ratio (GDR) in five SDI quintiles in comparison to global trend and in different age categories.

The analysis of QCI in various age groups in 2017 revealed variations and changing trends. Age groups between 75 and 89 had the lowest QCI scores (70–75), whereas those between 35 and 49 years scored highest (90). Between the ages of 40 and 60 years, we saw a steady‐state of QCI. However, between the ages of 60 and 80 years, there was a decline. The lowest scores were seen at about the age of 80. QCI increased after the age of 80 years. Various SDI quintiles showed that the SDI of a region was directly proportional to its QCI score. High SDI regions scored higher than the global QCI. In contrast, low and low–middle regions scored lower than the global QCI (Figure 3A).

The global TC incidence rate was higher in higher age groups, and once over the age of 75–85 years, it ascended sharply. A similar pattern was seen in SDI regions, with higher SDIs holding higher positions in comparison. Globally, the mortality rate started to rise after the age of 40–45 years. The highest death rates were seen in the under 80 years of age groups in low SDI regions.

### 3.4 Gender disparity ratio

The global trend of GDR in 2017 demonstrated that 20–40 years of age groups scored lower than one. Starting from the 5th decade of life, GDR started to rise to more than 1, and except for a subtle decline at about the age of 70, it remained higher than 1 in higher age groups. The lowest GDR was seen in 20–25 years age group (0.94), favoring better care in men. The highest GDR was among 60–65 years of age group, at about 1.06, suggesting better care in women. GDR patterns varied entirely in various SDI regions. For example, in low and low–middle SDI countries, the highest sex disparity was about 1.2 between 40 and 60. In middle and high–middle SDI countries, GDR increased after the age of 40 years. Among these regions, high SDI countries had equal care conditions among both genders, as the GDR score in all ages was just near one, ranging from 0.99 to 1.03 (Figure 3B).

**FIGURE 4:**
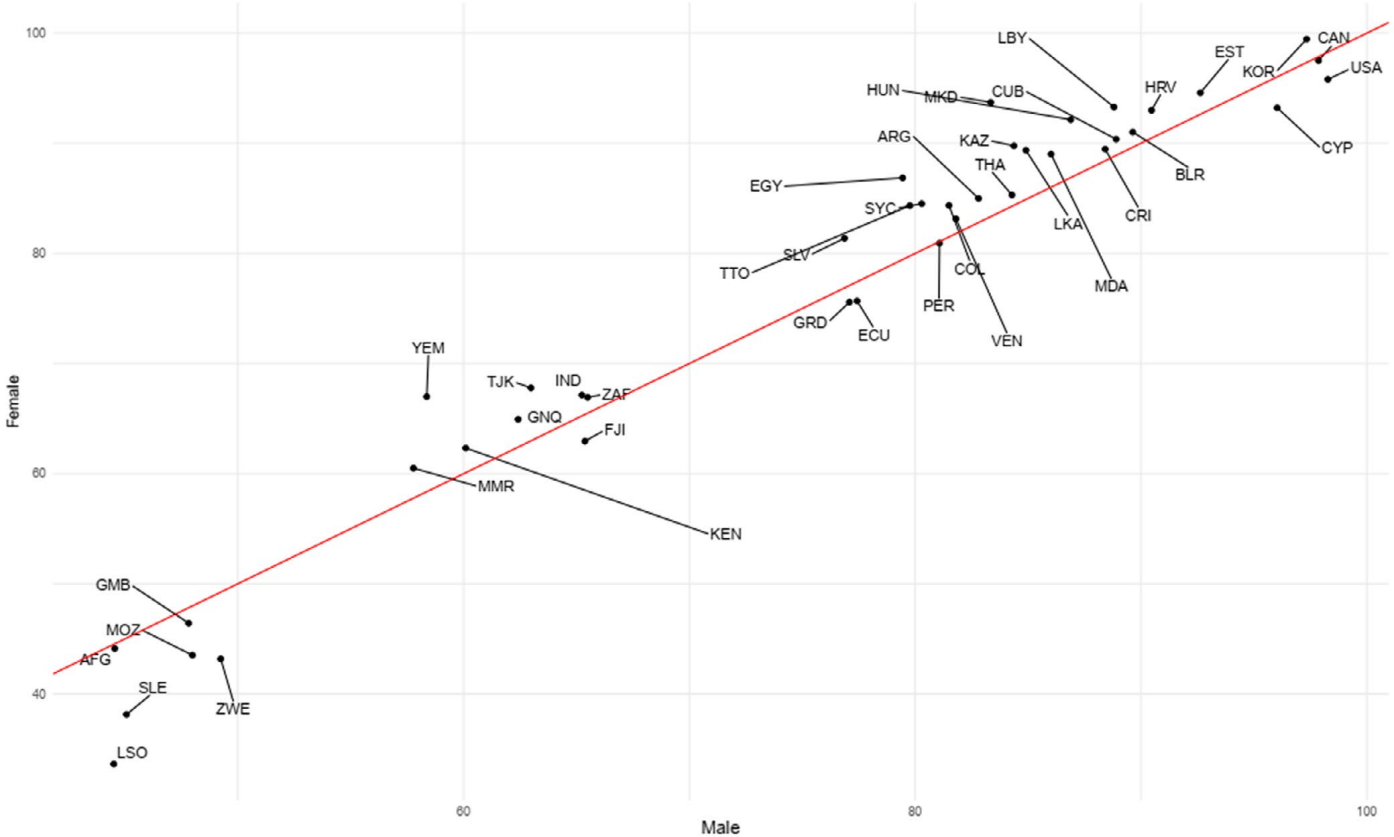
Comparison of disparity of estimated quality of care index (QCI) for both sexes in different countries.

Figure 1B shows GDR in different countries. The Central African Republic, Chad, and Somalia had the lowest ratios, while Ghana, Uzbekistan, and Bosnia and Herzegovina had the highest ratios, suggesting gender disparity in both cases. Countries that had near‐one ratio scores showed equal care, but this did not necessarily imply better care. Therefore, to truly compare these findings, as illustrated in Figure 4, countries such as Afghanistan and Gambia had a near‐one GDR, but QCI in both genders was very low. However, not only did countries like the United States of America, Canada, and South Korea have a near‐one GDR, but also high QCIs for both sexes.

In 1990 and 2017, the TC incidence rate had been 2–3 times higher in women (1990: 3.04 for women and 1.15 for men; 2017: 4.34 for women and 1.94 for men). Moreover, the mortality rates in these years had been about 1.5 times greater in females (1990: 0.66 for females and 0.41 for males; 2017: 0.57 for females and 0.47 for males).

## 4 DISCUSSION

We found that the incidence and burden of TC have increased significantly. The QOC was lower in developing countries but substantially higher in wealthy nations and developed regions. QOC was higher in younger adults. Global gender disparity was in favor of the female gender due to better care among women.

The interpretation of the primary epidemiologic indices of TC showed dramatic changes during the 28‐year study period. New diagnostic and screening strategies, increasing risk factors, and higher incidence of real malignant cases was among the various factors that contributed to the rapidly rising incidence of TC.^1^, ^33^, ^34^, ^35^, ^36^ Many evidences suggest that the overdiagnosis of TC is the main explanation of this expansion.^4^, ^33^ TC is prone to overdiagnosis mainly because it has an indolent progression and has low mortality rates. Introducing more sensitive ultrasound, computed tomography, ultrasound‐guided fine‐needle aspiration, and cytology investigations increased detection of many small and unknown subclinical TCs.^4^, ^37^ After introducing ultrasound in the diagnosis and screening of TC, a significant number of incidental thyroid nodules have been detected, and a large fraction of TCs are diagnosed by pathologic examination of such nodules.^33^

This study revealed higher incidence rates in higher SDI regions but higher mortality rates in lower SDI regions. In developed countries, a possible explanation for the higher incidence might be more exposure to environmental risk factors such as ionizing radiation and other carcinogens and more access to diagnostic tools. The lower mortality rates in these countries, however, are mainly the result of early detection, better healthcare access, and suitable treatment.^2^, ^13^, ^38^ In this study, the increase of TC burden was significant in every measure, including DALY, YLL, and YLD. The overall 10‐year survival rate for most treatable types of TC was estimated at 92%–98%, except for incurable types like anaplastic thyroid carcinoma. Nonetheless, TC patients often die of other causes rather than cancer.^39^, ^40^

The QCI helped us evaluate cancer care quality in different populations. The European region, the region of the Americas, and the Western Pacific region had higher than global QCI rates. In contrast, the Eastern Mediterranean region, South East Asia region, and African region gained lower scores. Also, high and high–middle SDI regions acquired higher scores than the other three regions. Among the 195 countries of GBD, the majority scored lower than the global score. Almost all countries with higher QCI were located in the abovementioned higher SDI regions.

We did a more precise inspection of regions and countries with higher QCI scores and discovered three major factors that could help better policymaking in TC care. First, most of these countries had TC screening and management guidelines and practiced based on them. For example, the guidelines for TC by the American Thyroid Association (ATA) and the European Society for Medical Oncology (ESMO) outline the most updated and efficient clinical guidelines.^41^, ^42^ Second, certain countries such as Italy and Singapore that scored high QCIs have developed consensuses on TC. Italy's consensus on the diagnosis and treatment of TC is a joint statement of six Italian societies engaged in the treatment of TC patients.^43^ Third, countries with higher QOC had established TC registries and had predicted the growth of TC. For instance, The Australian & New Zealand Thyroid Cancer Registry (ANZTCR) was established in 2017 to improve TC patients' outcomes and QOC.^44^ Studies in Italy and Lebanon in the early 2000s investigated TC incidence trends and projections, which have had better QOC.^45^, ^46^

Evaluating the barriers toward achieving sufficient QOC in these patients was also helpful. A major problem was related to the coordination and communication of cancer caregivers with one another and their patients.^47^ Other obstacles to the health systems were legal and insurance issues regarding patient care.^16^ One previous survey on an oncology society of patients described three major categories of barriers to access to quality healthcare for cancer patients; health system, social/environmental, and individual barriers. Of all the subcategories, inadequate health insurance and the inability to pay treatment costs were the more prominent problems. Therefore, providing suitable insurance and costs coverage can promote QOC in these patients.^48^

The inspection of TC QCI in various age groups revealed an overall higher QOC in younger adults, while older people received lower QOC. One possible cause may have been the poorer prognosis of TC in older age groups because of their weakened immune system, multimorbidity, and higher all‐cause mortality.^49^, ^50^ Another explanation for the lower QOC in older people could have been the rapid population aging process and paucity of informed and skilled caregivers in most countries.^51^

Moreover, overall better care was seen in favor of women in higher SDI regions, whereas men received better care in lower SDI regions. Standardized incidence and mortality rates were revealed to be higher among women. Three significant theories were suggested for these disparities in men and women. First, women go through more thyroid examinations because of a higher incidence of nonmalignant thyroid disorders in their medical history. Second, behavioral variations between genders make women seek medical evaluations in earlier stages. They also get more involved during medical visits. The final and the third theory suggested for a reason behind these disparities is the biological differences between males and females.^12^, ^52^, ^53^

One of this study's limitations was the absence of an evaluation of racial and ethnic disparities. This was mainly due to the lack of information regarding TC patients in the GBD study. Other possible limitations were the deficiencies in the data registry of the countries in the IHME‐GBD data sets that could have affected our results. However, IHME tends to collect complete data, and in regions with insufficient information, estimates indices by using comprehensive statistical models. Unfortunately, calculating the confidence interval for all estimated indices and significant values in this study was impossible due to limited statistical and computational resources. One of this study's strengths was evaluating TC QOC in different dimensions since the lack of previous researches on this topic was a problem. Understanding the real trends of TC epidemiology needs further observations to be continued longitudinally and interpreted over time to estimate more accurate findings. Further similar approaches can help to emphasize the importance of QOC.

## 5 CONCLUSIONS

In this study, QCI was created and introduced as a practical means to assess and compare cancer care quality on different scales. We demonstrated that the development status was directly associated with QCI and gender/age equity. Also, we suggest that QCI can be used as a useful measure to study the QOC in other cancers and diseases. Considering the epidemiologic aspects and inequities of TC care, more efficient public healthcare planning is required.

## CONFLICT OF INTEREST

The authors declare no conflict of interests. All of the authors approved the final draft.

## ETHICAL STATEMENT

No individual data was reported in this paper and the information is based on an aggregated pre‐existing online data (available on https://vizhub.healthdata.org/gbd‐compare/). No ethical committee approval was sought for this survey.

## DECLARATION

None of the authors listed on the manuscript are employed by a government agency that has a primary function other than research and/or education. Also, none of the authors are submitting this manuscript as an official representative or on behalf of the government.

## Supporting information

Table S1

## Data Availability

The data that support the findings of this study are available from the corresponding author upon reasonable request.
